# Valuation of preference-based measures: can existing preference data be used to generate better estimates?

**DOI:** 10.1186/s12955-018-0945-4

**Published:** 2018-06-05

**Authors:** Samer A. Kharroubi

**Affiliations:** Department of Nutrition and Food Sciences, Faculty of Agricultural and Food Sciences, American University of Beirut, Beirut, Lebanon

**Keywords:** Preference-based health measure, Non-parametric Bayesian methods, Time trade-off, EQ-5D

## Abstract

**Background:**

Experimental studies to develop valuations of health state descriptive systems like EQ-5D or SF-6D need to be conducted in different countries, because social and cultural differences are likely to lead to systematically different valuations. There is a scope utilize the evidence in one country to help with the design and the analysis of a study in another, for this to enable the generation of utility estimates of the second country much more precisely than would have been possible when collecting and analyzing the country’s data alone.

**Methods:**

We analyze SF-6D valuation data elicited from representative samples corresponding to the Hong Kong (HK) and United Kingdom (UK) general adult populations through the use of the standard gamble technique to value 197 and 249 health states respectively. We apply a nonparametric Bayesian model to estimate a HK value set using the UK dataset as informative prior to improve its estimation. Estimates are compared to a HK value set estimated using HK values alone using mean predictions and root mean square error.

**Results:**

The novel method of modelling utility functions permitted the UK valuations to contribute significant prior information to the Hong Kong analysis. The results suggest that using HK data alongside the existing UK data produces HK utility estimates better than using the HK study data by itself.

**Conclusion:**

The promising results suggest that existing preference data could be combined with valuation study in a new country to generate preference weights, making own country value sets more achievable for low and middle income countries. Further research is encouraged.

## Background

Health resource allocation is becoming increasingly important in an economic climate of increasing demands on healthcare systems with constrained budgets. Economic evaluation using cost-utility analysis has become widely popular technique internationally to inform resource allocation decisions. Cost-utility analysis measures benefits using Quality Adjusted Life Years (QALYs), a measure that multiples a quality adjustment for health by the duration of that state of health [1]. The quality adjustment weight is generated using utility values where 1 denotes full health and 0 denotes dead, and is most often generated using an existing preference-based measure. Such a measure consists of a classification system used to describe health (patients report their own health and this is assigned to a health state using a classification system) and a value set that generates a utility value for every health state defined by the classification system.

Among the large number of currently available preference-based measures of health-related quality of life (HRQoL) are the generic EuroQol five dimensional (EQ-5D) questionnaire [2], health utilities index 2 (HUI2) and 3 [3, 4], Assessment of Quality of Life (AQoL) [5], Quality of Well-being scale (QWB) [6], and the six-dimensional health state short form (derived from short-form 36 health survey) (SF- 6D) [7], though there are an increasing number of condition-specific measures available [8].

There is now an increasing number of datasets of preference data, where preferences have been elicited for the same measure for different countries. Kharroubi et al. [9] use a novel nonparametric Bayesian approach to model the disparities between the United States (US) and UK which is simpler, better fitting and more appropriate for the data than the previously adopted conventional parametric model of Johnson et al. [10]. Such an approach has also been applied to the joint UK-Hong Kong and UK-Japan SF-6D data set ([11], [12]). The nonparametric Bayesian model offers a major added advantage as it permits the utilization of findings of country 1 to improve those of country 2, and as such generated utility estimates of the second country will be more precise than would have been the case if that country’s data was collected and analyzed on its own.

There are two distinct ways in which such a model may be useful. In the existence of large quantity of data pertaining to two countries, good estimates of population utility functions corresponding to each country can be generated through the analysis of data from each country on its own (using the model of [13]) and this is the best option. However, in case where a significant quantity of data is available in one country but limited in another, there is a scope to borrow strength from country 1 in an effort to obtain better population utility estimates for the second country than those generated when analyzing that second country’s data on its own.

Recently, Kharroubi [14, 15] developed a modified nonparametric Bayesian statistical method that permits the utilization of evidence from one country as substantial prior information for a study in another, and employed this method in the analysis of a valuation study for EQ-5D in US using the already existing UK data. Crucial assumption underlying this analysis was that preferences of the UK population are in essence the same as those of the US in addition to that both countries have plenty of data. However, different countries have different population compositions, work, cultures and language. These can all impact on the relative values given to different dimensions of health (for example, self-care and anxiety/depression) as well as where on the 1-0 full health-dead scale each health state lies.

The present paper seeks to explore the use of such a model in the context of smaller countries with different cultures. This is explored using a case study for SF-6D HK and UK data, where the health states valued in the HK valuation study are modelled using the already existing UK dataset, and the estimates are compared to the estimates generated modelling HK data alone. It should be noted that this method was used to model the US/UK data (the Kharroubi et al. [14, 15] articles describe this at length), and as such the method given in this article is a replication of that method. Hence, though it does not present new methodological developments, it further accentuates the key point made in the Kharroubi et al. [14, 15] articles, i.e. the good performance of the new modelling approach.

First, SF-6D valuation surveys along with employed data corresponding to UK and HK are summarized here. Second the Bayesian non-parametric model is described and third the results are presented. Finally, the results are discussed, including limitations and suggestions of possible future outlooks.

## Methods

### The SF-6D

The SF-6D includes six health dimensions: *physical functioning, role limitation, social functioning, bodily pain, mental health* and *vitality,* each with between four and six levels [7]. Through the selection of one level from each dimension, physical functioning being the first and vitality being the last, an SF-6D health state is defined. Different combinations result in 18,000 possible health states, which are associated with a six-digit descriptor ranging from 111,111 representing full health and 645,655 representing the worst possible state called “the pits”.

### The valuation survey and data set

#### UK

A sample of 249 health states is described through the SF-6D and then valued by a representative sample of the UK population (*n* = 836). Selection methods of respondents along with health states are discussed elsewhere [7]. All the selected respondents have been asked to rank and value six health states according to the McMaster ‘ping pong’ variant of the standard gamble (SG) technique. Accordingly, each of the five SF-6D health states was valued against the perfect health state and against the “pits” by the respondents. As for the sixth question, it consisted of valuing the “pits” by determining whether they perceived it as worse or better than death by considering one of the following choices: (i) the certain prospect of being in the “pits” state and the uncertain prospect of full health or immediate death; or (ii) the certain prospect of death and the uncertain prospect of full health or the “pits” state [16]. Negative values were bounded at − 1, and they designate the states value as worse than death [17]. Then, the other 5 health states were chained onto the zero to one scale, where 0 s designates the perceived equivalent to being dead, and 1 corresponds to perfect health [7]. As such, the dependent variables (*y*) in the models below correspond to the adjusted SG values.

Of the original 836 respondents, a total of 225 respondents had to be excluded for several reasons. For instance, 130 respondents failed to value the “pits” state; consequently, the corresponding data couldn’t be processed any further [10]. Of the total 611 included respondents, 148 missing values from 117 respondents were present thereby resulting in a total of 3518 observed SG valuations across the 249 health states. Details pertaining to the valuation of the 249 SF-6D UK health states can be found in [7].

#### Hong Kong

The HK study comprised of a sample of 197 health states (selected according to the UK procedures) which were valued using the same valuation procedures as those in the UK study [18]. Each respondent was asked to rank and value eight health states, and the interview procedure was modelled on the basis of that in the UK study.

Out of the original 641 respondents, a total of 59 respondents were disqualified from the analysis according to the same exclusion conditions as in the UK study [6] leaving 582 respondents’ data for analysis. Each of the 582 respondents made 8 SG valuations, giving 4596 valuations. Of these, 60 missing health state values were present and so 4596 observed SG valuations across 197 health states were finally included in the analysis. Details pertaining to the valuation of the 197 SF-6D HK health states can be found in [18].

### Modelling

The modelling approach is described in Kharroubi [14], where a nonparametric Bayesian model was employed in the modelling of the US EQ-5D dataset using the already existing UK dataset as informative prior. In this article, we follow on from its work to examine whether the adoption of HK health states, while drawing extra information from the UK data, generates better estimation than analyzing the HK sample by itself. The estimates are compared using different prediction criterion, including predicted versus actual mean health states valuations, mean predicted error and root mean square error.

Kharroubi [14] propose the following model

1 $$ {y}_{ij}=1-{\alpha}_j\left\{1-u\left({\mathbf{x}}_{ij}\right)\right\}+{\varepsilon}_{ij} $$

Where, for *i* = 1,2,…,*I*_*j*_ and *j* = 1,2,…,*J, x*_*ij*_ is the *i-*th health state valued by the respondent *j* in the HK experiment*, y*_*ij*_ is the respondent *j*’s time trade-off (TTO) valuation for that health state *i*, *α*_*j*_ is a term to allow for individual characteristics of respondent *j* and *ε*_*ij*_ is a random error term. Let ***t***_*j*_ be a vector of covariates representing individual characteristics of respondent *j*, Kharroubi [14] propose the following distributions:

$$ {\alpha}_j\sim LN\left({t}_j^T\gamma, {\tau}^2\right)\ \mathrm{and}\ {\varepsilon}_{ij}\sim N\left(0,{\upsilon}^2\right). $$

where ***γ*** is the vector of coefficients for the covariates and *τ* ^*2*^ and *v*^*2*^ are further parameters to be estimated.

We next let ***u***(**x**) and *u*_*UK*_(**x**) be the utility functions for health state **x** valued in the HK and UK experiments respectively, Kharroubi [14] then model the prior distribution for ***u***(**x**) as multivariate normal with mean defined as

2 $$ E\left(u\left(\mathbf{x}\right)\right)=E\left({u}_{UK}\left(\mathbf{x}\right)\right)+\gamma +{\beta}^{\prime}\mathbf{x} $$

and variance-covariance matrix

3 $$ \operatorname{cov}\left({u}_{UK}\left(\mathbf{x}\right),{u}_{UK}\left({\mathbf{x}}^{\prime}\right)\right)+{\sigma}^2\mathrm{c}\left(\mathbf{x},{\mathbf{x}}^{\prime}\right) $$

where *E*(*u*_*UK*_(**x**)) is the expected value of the utility of health state x and cov(*u*_*UK*_(**x**), *u*_*UK*_(**x**′)) is the variance-covariance matrix between *u*_*UK*_(**x**) and *u*_*UK*_(**x**′) for two different states **x** and **x**′ in the UK experiment, both of which are readily available from the analysis of the UK study.

Given Eqs. 2 and 3, note that **x** represents a vector consisting of discrete levels on each of the six health dimensions and *γ*, *β* and *σ*^2^ are unknown parameters. If follows from Kharroubi [14] that the mean function of ***u***(**x**) represents a prior expectation that the utility will be approximately a simple additive linear function of the dimension level in **x**. Additionally, the true function is allowed to deviate around this mean according to its multivariate normal distribution, and so it can as a result assume any form. It is in this sense that the Bayesian model is described as nonparametric. Furthermore, there seem to be a high correlation c(**x,x**′) between *u*(**x**) and *u*(**x**′) when **x** and **x**′ are close enough, and is given by

4 $$ \mathrm{c}\left(\mathbf{x},{\mathbf{x}}^{\prime}\right)=\exp \left\{-\sum {b}_d{\left({x}_d-{x}_d^{\prime}\right)}^2\right\} $$

where *b*_*d*_ is a roughness parameter in the dimension *d* that controls the extent to which the true utility function is anticipated to adhere to a linear form in a dimension *d*. It is to be noted that many other choices have been made for this covariance matrix; see for example [19] or [20], but the resulting estimates are not generally sensitive to the change of this function. However, the proposed form is appropriate here [13]. See Kharroubi et al. [14] for more details on this.

Finally, it is to be noted that the novel method of modelling utility function *u*(**x**), represented by adding the two terms *E*(*u*_*UK*_(**x**)) and cov(*u*_*UK*_(**x**), *u*_*UK*_(**x**′)) in Eqs. 2 and 3, allows the already existing UK evidence to contribute significant prior knowledge to the HK study. In other words, the posterior density of the UK utility function was treated as a prior density to analyse the new study in the HK.

Full theory of the Bayesian approach here is discussed in Kharroubi [14]. Programs to undertake the Bayesian approach were written in Matlab and are available on request.

## Results

The new modelling approach is now applied to the analysis of SF-6D HK study using the previously existing UK study (to be indicated by HK/UK model hereinafter). From a Bayesian prospective, the old posterior contains all that we know before seeing the new data, and so becomes the new prior distribution. Thus for our analysis, the posterior of the UK utility function becomes our prior for the analysis of the HK study. The estimates are compared to those estimated using the HK data excluding the UK data (to be indicated by HK model hereinafter) using different prediction criterion, including predicted versus actual mean health states valuations, mean predicted error, root mean square error along with the Bland-Altman agreement plots [21].

**Fig. 1:**
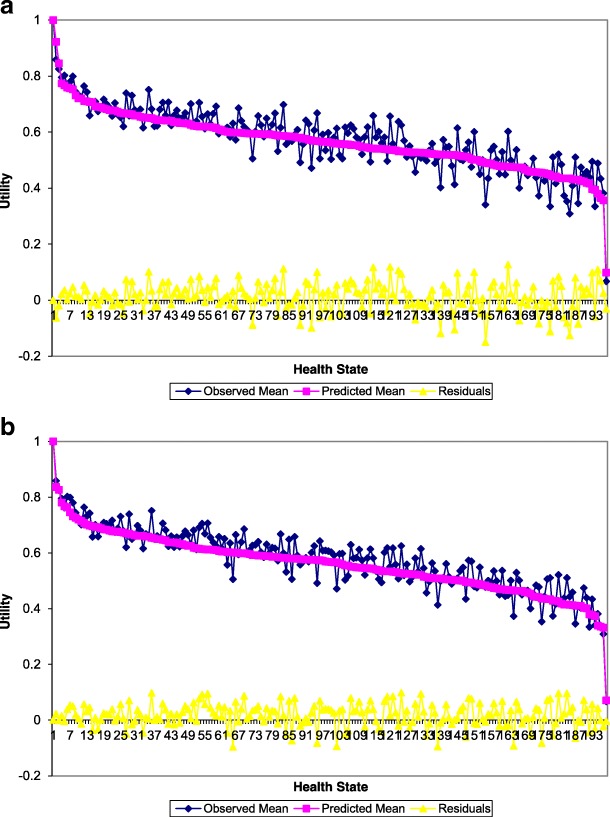
Sample mean and predicted health states valuations for **a** the HK model and **b** the HK/UK model

Figure 1 shows the HK predicted and observed mean valuations corresponding to the 197 health states evaluated in the sample along with the perfect health, sorted via the predicted valuations. Figure 1a shows the predicted (squared line) and actual (diamond marked line) mean valuations using the HK model. The line marked with triangles denotes the errors computed based on the difference between the two valuations. Figure 1b shows the corresponding results obtained using HK/UK model. Based on the plots it is apparent that the estimates of the HK/UK utilities for the various SF-6D health states are much more precise than those corresponding to the HK only results. These plots also reveal the HK model tends to under predict at low health state values (meaning the poor health states). However, this is not the case for the HK/UK model. Additionally, the plots suggest that the variations of the predictions are larger and so a high fluctuation and non-steady trend of the difference line, so this suggests that the HK/UK model is less susceptible to systematic bias.

**Fig. 2:**
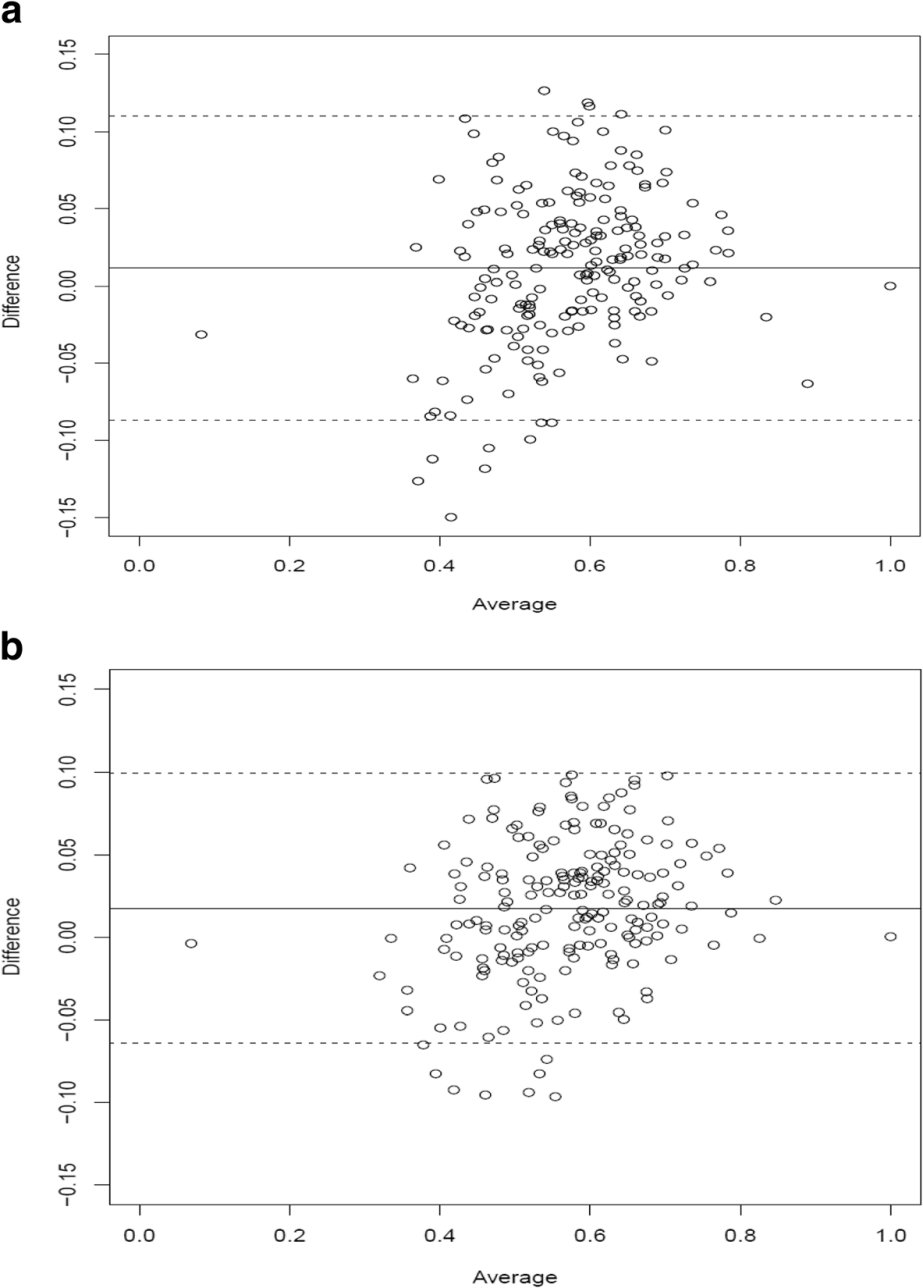
Bland-Altman agreement plots for **a** the HK model and **b** the HK/UK model

Figure 2a and b depict the Bland–Altman agreement plots for HK and HK/UK models. In this context, the difference between the observed and predicted mean valuations is plotted against the mean of the difference (or the average bias). The solid line corresponds to the mean bias, whereas the dotted lines depict the 95% limits of agreement. For better visual judgment of how good the two valuations agree, the 95% limits-of-agreement lines are drawn. The narrower the range between these two limits, the better the agreement is. When comparing these two figures, we see that the HK/UK model reveals a better agreement as the length of the 95% limits of agreement is 0.163, i.e. narrower than that of the HK model of length 0.197. Additionally, the difference in mean bias between the two models is also obvious, with values of 0.0116 for the HK/UK model and 0.0175 for the HK model. Moreover, the differences standard deviation corresponding to the HK/UK model is much smaller (0.0416) as compared to that corresponding to the HK model (0.0503), thereby vindicating the variations of the differences in Fig. 2a. On the other hand, the HK/UK model differences are well validated as observed in Fig. 2b.

Table 1 provides the inferences for the utilities of the 197 states evaluated in the study along with the perfect health. Table 1 displays the actual mean, the standard error corresponding to each health state for both models. The results for the population utilities from the UK that were treated as prior information in the HK/UK model are also provided. As depicted all through the 197 health states (excluding the perfect health state) presented in Table 1, it is evident that the HK/UK model has a better predictive performance compared to the HK model overall, and as a results it has a root mean square error (RMSE) of 0.045 whereas the HK model has an RMSE of 0. 051.

**Table 1:** Posterior inferences for utilities of the 197 health states valued in the empirical survey along with the perfect health

State X	Observed mean	UK results		HK Model		HK/UK Model	
		Posterior mean	Posterior SD	Posterior mean	Posterior SD	Posterior mean	Posterior SD
111111	1	1	0	1	0	1	0
111621	0.6492	0.7482	0.0345	0.6322	0.029	0.6652	0.0267
111645	0.5055	0.6169	0.0586	0.5568	0.0281	0.5384	0.0316
112153	0.6519	0.691	0.0577	0.6681	0.0293	0.6524	0.0326
112455	0.6777	0.6078	0.0515	0.635	0.0298	0.6274	0.0325
112613	0.7305	0.7049	0.0612	0.6636	0.0321	0.674	0.0364
112651	0.6288	0.6342	0.0614	0.6182	0.0335	0.5919	0.0366
113141	0.8022	0.7581	0.0643	0.7665	0.0287	0.7633	0.0322
113352	0.7441	0.6776	0.0523	0.7304	0.0262	0.7253	0.0282
113411	0.7324	0.7284	0.031	0.7208	0.0287	0.701	0.0255
113615	0.6685	0.6415	0.0677	0.6308	0.0334	0.6125	0.0401
113634	0.64	0.6017	0.0535	0.5971	0.0305	0.5902	0.0352
114631	0.7057	0.6754	0.056	0.6418	0.0343	0.6467	0.0376
115131	0.7243	0.7695	0.0704	0.7203	0.029	0.7193	0.0317
115211	0.7951	0.7738	0.0561	0.7739	0.0309	0.7802	0.0327
115251	0.6223	0.7148	0.074	0.6427	0.0287	0.6329	0.0318
115314	0.6788	0.779	0.0545	0.6585	0.0312	0.6731	0.0347
115355	0.5346	0.5859	0.0563	0.5649	0.0306	0.5396	0.0347
115432	0.6201	0.7091	0.06	0.6673	0.0265	0.6696	0.0287
115653	0.5728	0.5652	0.0561	0.5189	0.0308	0.494	0.0325
121212	0.8253	0.8275	0.0261	0.8452	0.0249	0.8259	0.0233
122233	0.6894	0.7475	0.034	0.6882	0.0273	0.6888	0.0273
122425	0.704	0.6784	0.0353	0.6764	0.0262	0.683	0.0268
124125	0.612	0.7292	0.0475	0.6194	0.0279	0.6158	0.029
125143	0.6505	0.6892	0.053	0.6515	0.0284	0.649	0.0301
125625	0.5478	0.5779	0.0663	0.5187	0.0326	0.4992	0.0374
131151	0.7621	0.7402	0.0725	0.7591	0.0293	0.767	0.0331
131331	0.7638	0.7629	0.0522	0.7103	0.0292	0.7071	0.0326
131542	0.7067	0.6181	0.0304	0.6407	0.0276	0.6118	0.0269
131555	0.5327	0.5832	0.0544	0.5345	0.0313	0.521	0.0349
132524	0.5983	0.6574	0.037	0.5944	0.0265	0.5819	0.0274
133132	0.7425	0.6942	0.0343	0.7093	0.0266	0.6978	0.0263
135155	0.6251	0.5947	0.0639	0.5928	0.032	0.5747	0.0365
135312	0.7	0.6992	0.0488	0.6814	0.0272	0.6802	0.0283
135435	0.655	0.5664	0.063	0.6194	0.0291	0.6114	0.0327
135633	0.5085	0.5848	0.0613	0.5205	0.0304	0.5014	0.0353
141215	0.7089	0.7227	0.0698	0.6916	0.0303	0.6843	0.035
142113	0.6585	0.6911	0.0543	0.7071	0.0277	0.6917	0.0294
142154	0.6821	0.6844	0.0373	0.6494	0.0281	0.6444	0.0274
142335	0.6654	0.6536	0.0529	0.6164	0.0294	0.6	0.0332
143641	0.5733	0.6151	0.0506	0.5495	0.0329	0.5461	0.0349
143654	0.5028	0.5487	0.0585	0.5145	0.0306	0.5018	0.0341
144341	0.5565	0.72	0.0279	0.5856	0.0275	0.6026	0.0243
144455	0.5676	0.5356	0.0643	0.5839	0.0299	0.5768	0.0352
144613	0.6916	0.6501	0.0583	0.6136	0.0334	0.6147	0.0385
145515	0.5903	0.617	0.0735	0.5834	0.0322	0.5786	0.0371
145621	0.6093	0.6233	0.0645	0.6026	0.0315	0.5952	0.0361
145645	0.5814	0.5077	0.0715	0.5391	0.0316	0.5427	0.0347
145652	0.5291	0.5334	0.0678	0.4771	0.0328	0.4634	0.0385
211111	0.8584	0.9197	0.0215	0.9219	0.0181	0.836	0.0205
211251	0.6738	0.7049	0.0631	0.6901	0.0278	0.6763	0.0299
211615	0.6206	0.6781	0.0713	0.6458	0.0307	0.6372	0.0357
211633	0.7051	0.6622	0.0527	0.62	0.0319	0.6129	0.0354
212145	0.6188	0.6927	0.0446	0.5961	0.0277	0.5851	0.0279
213323	0.6571	0.7761	0.0296	0.6767	0.0228	0.6946	0.0226
214435	0.5943	0.6291	0.0401	0.6097	0.0272	0.5997	0.0293
221452	0.6627	0.6237	0.0459	0.6727	0.0261	0.6585	0.0279
224612	0.6385	0.6256	0.0392	0.6217	0.0288	0.5986	0.0298
232111	0.7796	0.6987	0.0377	0.7564	0.0273	0.7304	0.0264
235224	0.6506	0.6486	0.0335	0.632	0.0271	0.6042	0.0274
241135	0.5824	0.702	0.0601	0.5989	0.0297	0.5758	0.0327
241531	0.6643	0.702	0.0352	0.6189	0.0275	0.625	0.027
243433	0.5053	0.702	0.0351	0.5941	0.0259	0.6017	0.026
243615	0.5913	0.6257	0.0643	0.5651	0.0301	0.5659	0.0332
244353	0.6976	0.61	0.0413	0.5863	0.0307	0.6613	0.034
311654	0.6581	0.5391	0.0452	0.5414	0.0336	0.6066	0.0365
312332	0.701	0.7472	0.0285	0.7068	0.0241	0.7146	0.0229
315123	0.5582	0.8043	0.0542	0.5361	0.0312	0.531	0.0325
315235	0.6161	0.7018	0.0542	0.5866	0.0291	0.585	0.0329
315341	0.6486	0.7105	0.0618	0.592	0.0307	0.5796	0.0359
315515	0.6064	0.6642	0.0363	0.5686	0.0295	0.5675	0.029
321122	0.7987	0.7638	0.0266	0.7525	0.0256	0.7451	0.0239
323644	0.4377	0.5362	0.0287	0.4567	0.0298	0.4148	0.0269
324155	0.6248	0.6015	0.0479	0.5536	0.0334	0.6095	0.0374
325433	0.5685	0.6875	0.0451	0.5845	0.0262	0.5754	0.0294
331115	0.6584	0.7288	0.0598	0.6649	0.0275	0.6621	0.0297
332411	0.6152	0.7217	0.0376	0.6523	0.0246	0.6607	0.0246
333135	0.631	0.6657	0.0349	0.6219	0.0284	0.6249	0.0278
333455	0.6131	0.5504	0.0448	0.5588	0.0315	0.5438	0.0352
334251	0.5031	0.6761	0.0532	0.5621	0.0266	0.5547	0.0289
341123	0.7389	0.7009	0.0393	0.6653	0.0296	0.6685	0.0289
341251	0.6023	0.679	0.067	0.6064	0.0307	0.59	0.0339
341414	0.7513	0.6751	0.0574	0.6503	0.0317	0.6535	0.0363
341634	0.5209	0.6174	0.0409	0.5463	0.0297	0.5454	0.0295
341651	0.6015	0.5904	0.0633	0.5401	0.0325	0.5338	0.0352
342613	0.6672	0.6342	0.0526	0.589	0.0299	0.583	0.0334
343425	0.6307	0.6443	0.0405	0.5981	0.0284	0.5881	0.0303
344633	0.5002	0.6267	0.0395	0.493	0.0299	0.4787	0.0319
345153	0.4966	0.5875	0.0578	0.5378	0.0287	0.5242	0.0311
345355	0.4751	0.5101	0.0557	0.5036	0.0285	0.4895	0.0321
345411	0.6347	0.6506	0.0533	0.6302	0.0303	0.6019	0.0344
345535	0.6564	0.5436	0.0561	0.5377	0.0317	0.6356	0.036
345553	0.4538	0.5276	0.0527	0.4548	0.0307	0.4436	0.0345
411612	0.6595	0.6584	0.0634	0.6355	0.0305	0.6314	0.0332
412152	0.608	0.6558	0.0371	0.5804	0.0254	0.5715	0.0256
413212	0.6879	0.7402	0.0485	0.678	0.028	0.6757	0.0298
414355	0.5976	0.6335	0.0478	0.5634	0.0294	0.5588	0.0317
414522	0.7007	0.6612	0.0301	0.626	0.0303	0.6929	0.0299
415115	0.6264	0.7271	0.0617	0.5911	0.0321	0.592	0.0359
415313	0.5055	0.7889	0.0501	0.5672	0.0263	0.5795	0.0289
415453	0.5826	0.6483	0.0557	0.5458	0.0301	0.5477	0.0328
415651	0.5347	0.5696	0.069	0.4882	0.0334	0.474	0.0377
415655	0.4739	0.5087	0.0623	0.4259	0.0347	0.4026	0.0396
421314	0.6607	0.6689	0.0368	0.658	0.0253	0.6495	0.0261
421455	0.4016	0.6127	0.0383	0.5198	0.0275	0.4093	0.0286
421641	0.6118	0.635	0.0577	0.5533	0.0316	0.5464	0.0354
423435	0.6172	0.5985	0.0435	0.5564	0.0297	0.5321	0.0338
423615	0.6373	0.5506	0.0581	0.5312	0.0323	0.6111	0.038
425131	0.5312	0.6771	0.0551	0.5876	0.0253	0.5817	0.0273
431443	0.5838	0.638	0.0339	0.5927	0.0261	0.5889	0.0261
432621	0.6423	0.6468	0.0487	0.5754	0.0292	0.5735	0.0337
434211	0.6601	0.7068	0.0466	0.6409	0.0302	0.6375	0.0327
435335	0.6579	0.57	0.0499	0.5929	0.0295	0.5788	0.0337
441255	0.5133	0.5918	0.0522	0.5271	0.0324	0.5042	0.0346
441331	0.557	0.7049	0.0434	0.5765	0.0277	0.5772	0.0282
441615	0.4883	0.5871	0.0633	0.5209	0.0303	0.5033	0.0334
442655	0.353	0.5227	0.0536	0.4346	0.0301	0.4359	0.0327
443215	0.5719	0.6548	0.0352	0.5981	0.0272	0.5845	0.0267
443652	0.4431	0.5548	0.0564	0.4242	0.0312	0.4127	0.0353
444611	0.6854	0.6028	0.0592	0.5974	0.0312	0.5983	0.0345
445145	0.3405	0.552	0.0525	0.4903	0.0273	0.3726	0.0307
445233	0.4914	0.6384	0.0434	0.5801	0.0267	0.5741	0.0282
445615	0.4775	0.5487	0.0653	0.4665	0.0327	0.4409	0.0363
445641	0.5364	0.5241	0.0641	0.4739	0.033	0.4687	0.0378
511114	0.6239	0.6993	0.0379	0.64	0.0281	0.6376	0.0276
511435	0.6804	0.654	0.0546	0.6422	0.0298	0.6613	0.0306
511615	0.5991	0.5818	0.0725	0.5918	0.0313	0.5879	0.0372
511633	0.5805	0.5918	0.0611	0.5599	0.0298	0.5497	0.0349
512242	0.6013	0.6906	0.0324	0.5932	0.0255	0.5972	0.0261
513654	0.4584	0.5474	0.0525	0.4182	0.0328	0.4126	0.0361
515155	0.6677	0.5927	0.0618	0.5675	0.0348	0.6586	0.0395
522321	0.7164	0.6846	0.0324	0.6844	0.0264	0.6776	0.026
523551	0.6141	0.6201	0.0471	0.5167	0.0314	0.5206	0.0334
531635	0.5015	0.5323	0.0345	0.5003	0.0292	0.4633	0.0299
533415	0.5342	0.5848	0.0533	0.5228	0.0288	0.5086	0.0329
534113	0.5076	0.7106	0.0437	0.5266	0.0283	0.528	0.0293
541451	0.5194	0.6153	0.0626	0.5266	0.0286	0.5256	0.0325
543533	0.4771	0.674	0.0365	0.4745	0.0293	0.4834	0.0303
545115	0.5171	0.6074	0.0662	0.5582	0.0295	0.5545	0.0325
545151	0.5136	0.5474	0.0686	0.5256	0.0291	0.5225	0.0329
545353	0.5103	0.5243	0.0492	0.4303	0.0326	0.4147	0.0358
545422	0.6088	0.6351	0.0322	0.5954	0.0253	0.5688	0.0276
611154	0.5961	0.658	0.0636	0.5557	0.0329	0.5629	0.0354
611221	0.681	0.6667	0.0521	0.654	0.0319	0.6183	0.0344
611432	0.4712	0.6454	0.05	0.5706	0.0267	0.5654	0.0281
611454	0.3346	0.6146	0.0608	0.4466	0.0286	0.3353	0.0316
611621	0.5816	0.6112	0.0699	0.5529	0.0319	0.5447	0.0349
611645	0.4649	0.5249	0.0688	0.4731	0.031	0.4577	0.0354
611652	0.5207	0.5638	0.0616	0.437	0.034	0.4247	0.0383
612415	0.4566	0.5872	0.0632	0.5267	0.0292	0.5128	0.0327
613625	0.3453	0.5321	0.0646	0.4299	0.0298	0.4105	0.0345
614135	0.5587	0.6619	0.057	0.5224	0.0331	0.5247	0.0368
614434	0.4449	0.6497	0.0383	0.4615	0.0281	0.4682	0.0286
615253	0.6248	0.5737	0.0566	0.5308	0.0342	0.5269	0.0391
615315	0.5634	0.642	0.0628	0.5097	0.0334	0.5097	0.0369
615412	0.4129	0.6469	0.0544	0.5182	0.0282	0.5084	0.031
615451	0.4431	0.5666	0.0689	0.4499	0.0324	0.4353	0.0362
615455	0.4993	0.5404	0.0645	0.4753	0.0327	0.4723	0.0373
615614	0.4344	0.5683	0.0701	0.4885	0.0317	0.4952	0.0343
615631	0.5056	0.5247	0.0664	0.4574	0.0347	0.4338	0.0397
615653	0.381	0.5127	0.0681	0.356	0.0349	0.3388	0.039
621135	0.4934	0.6645	0.0605	0.5417	0.0291	0.535	0.032
622513	0.5108	0.5809	0.0392	0.529	0.0265	0.5069	0.0276
623155	0.4501	0.5938	0.0598	0.4784	0.0315	0.4631	0.0356
623353	0.4256	0.5718	0.043	0.4528	0.0318	0.4181	0.0346
624431	0.5694	0.5912	0.0379	0.53	0.0319	0.4933	0.033
624633	0.3082	0.551	0.0475	0.4345	0.0291	0.3316	0.0317
625141	0.5605	0.5561	0.0466	0.5398	0.0287	0.5047	0.0316
631315	0.5806	0.6157	0.0577	0.5403	0.0326	0.5223	0.0353
631333	0.6175	0.6386	0.0462	0.5443	0.0315	0.5336	0.0354
631355	0.4479	0.5823	0.0354	0.4765	0.0287	0.4666	0.0285
631632	0.4974	0.5525	0.0519	0.5252	0.0307	0.5102	0.0345
632615	0.5484	0.5202	0.0608	0.4831	0.0307	0.4872	0.0349
633122	0.4986	0.6515	0.0338	0.5131	0.0278	0.5084	0.0266
633535	0.3343	0.5378	0.0419	0.3942	0.0303	0.3791	0.0336
633653	0.4335	0.5395	0.0522	0.3644	0.0335	0.3776	0.0378
635611	0.4001	0.5522	0.0674	0.4736	0.0314	0.4538	0.0347
635651	0.4884	0.4829	0.0732	0.3799	0.0378	0.4841	0.044
641114	0.6165	0.6874	0.0653	0.6008	0.0313	0.6049	0.0335
641132	0.4794	0.6094	0.0542	0.5182	0.0312	0.4904	0.034
641154	0.545	0.5742	0.064	0.5188	0.0339	0.5143	0.0374
641211	0.6294	0.6567	0.0652	0.5718	0.0348	0.5505	0.0385
641654	0.4842	0.5225	0.0658	0.4347	0.0348	0.4418	0.0392
642151	0.5356	0.5776	0.0712	0.5119	0.0318	0.5009	0.0351
642313	0.5499	0.6834	0.0541	0.5278	0.0311	0.5332	0.0334
642453	0.5104	0.5844	0.054	0.4419	0.0326	0.4334	0.0366
642612	0.4496	0.5594	0.0336	0.4965	0.0282	0.4698	0.0289
642651	0.3731	0.5104	0.0707	0.4346	0.0308	0.4282	0.035
643,125	0.5007	0.6217	0.0556	0.4801	0.033	0.4661	0.0371
643143	0.463	0.6039	0.0531	0.4581	0.0321	0.4588	0.0333
644614	0.4387	0.5614	0.0573	0.4161	0.0321	0.4004	0.0376
644631	0.416	0.533	0.0623	0.4415	0.0313	0.4276	0.0352
645132	0.601	0.577	0.0517	0.5009	0.0352	0.5749	0.0384
645154	0.4948	0.5184	0.0614	0.396	0.0334	0.4765	0.0379
645235	0.3724	0.551	0.0592	0.4562	0.0299	0.4649	0.0339
645415	0.6023	0.5517	0.069	0.4761	0.0354	0.5666	0.0407
645441	0.4085	0.5106	0.0632	0.4309	0.0314	0.4094	0.0351
645655	0.067	0.3575	0.0186	0.0983	0.0226	0.0708	0.0251

*SD* Standard Deviation

Additionally, Table 1 indicates other noteworthy differences between the HK and HK/UK models. For the pits state, for instance, the HK model predicts a value of 0.0983 albeit the actual average for this state is 0.067, whereas the HK/UK model attains a value of 0.0708. Furthermore, the standard deviations corresponding to the HK/UK model are smaller as a result of using the UK results as priors thereby providing a better estimate. Differences in performance based on monotonicity are also apparent. Of the total 18,000 health states defined by the SF-6D descriptive system, 10,000 health states were sampled at random without replacement. In theory, there are 6–12 health states adjacent to each state of the 10,000 health. Then, as a result of selecting one health state at random from these 6-12 states, 10,000 adjacent pairs were obtained. Out of these 10,000 adjacent pairs, 20% display non-monotonicity in the HK model compared to 10% for the HK/UK model.

**Fig. 3:**
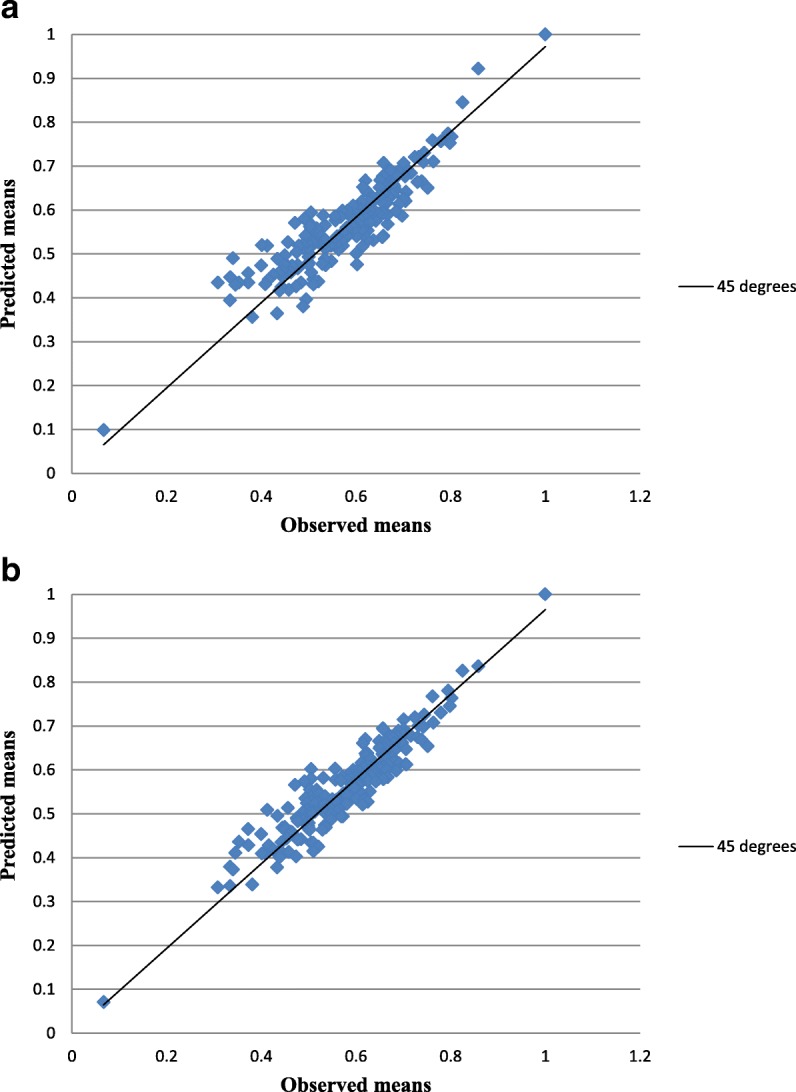
Sample mean and predicted health states valuations for **a** the HK model and **b** the HK/UK model

A more apparent presentation of the differences between the HK and HK/UK models is shown in Fig. 3, which depicts the fitted values corresponding to the HK model (Fig. 3a) and the HK/UK model (Fig. 3b) against the observed of the 198 health states, as well as the perfect predictions given by a 45° unity line (solid line). Theoretically, the fitted values from the two models are expected to lie roughly on the unity line. When comparing these two plots, it is clear from Fig. 3b that estimates from the HK/UK model tend to be more proximate to the perfect predictions line, in contrast to Fig. 3a, which depicts a larger scatter and the valuations deviate largely from the 45°theoretical line. As a result, we emphasize the fact that the HK/UK model provides predictions much more precisely than the HK model.

## Discussion

In this paper, we have applied a nonparametric Bayesian model to estimate the utility values of health states based on the SF-6D descriptive system. This model was undertaken in an effort to use the already existing information from one country to serve as an informative prior for a study in another. The methodology was applied to the HK SF-6D data set using the already available UK valuation, whereby the posterior of the UK utility function was used as a substantial prior to evaluate the new HK study. The method given here is a replication of that used in modelling the US/UK data (the Kharroubi et al. [14, 15] articles describe this fully). Hence, though it does not present new methodological developments, it further accentuates the key point made in the Kharroubi et al. [14, 15] articles, i.e. the good performance of the new modelling approach.

Crucial assumption underlying the US/UK analyses (Kharroubi et al. [14, 15]) was that preferences of the UK population are in essence the same as those of the US; in addition to that both countries have plenty of data. The novelty of the analysis presented here was to explore the use of new modelling in the context of smaller countries with different population compositions, work, cultures, language, all of which can impact on the relative values given to different dimensions of health (for example, self-care and anxiety/depression) as well as where on the 1-0 full health-dead scale each health state lies. This is explored using a case study for SF-6D HK and UK data, where the HK valuations are modelled using the already existing UK dataset and the estimates are compared to the estimates generated modelling HK data alone. It is shown that the new modelling of the utility function permitted the already existing UK dataset to contribute significant prior belief to the HK analysis, and for this to enable the generalisability of this approach by making use of experience in a European country to aid the analysis of a study in another Asian country. Consequently, much more precise estimates of the HK utilities corresponding to the various SF-6D health states were obtained using the HK/UK model than would have been the case if the data from HK study was used on its own, yet respect the inherent monotonicity of the underlying utility measure even further. Cautious model diagnostics affirm that the HK/UK model performs well and better than the HK model.

The nonparametric Bayesian model offers a major added advantage: in the existence of lots of data on one country and limited on another, it permits the utilization of results of country 1 to improve those of country 2, and as such generated utility estimates of the second country will be much more precise than would have been the case if that country’s data was collected and analyzed on its own. This in turn reduces the need for undertaking large surveys in every country using costly and more often time-consuming face to face interviews with techniques such as SG and TTO. To our knowledge, this concept hasn’t been investigated properly yet, but clearly it has a lot of potential value. Further research is underway to assess this.

Experimental studies to develop valuations of health state descriptive systems like EQ-5D, HUI or SF-6D need to be conducted in different countries and such work is costly and is potentially wasteful. The work presented here suggests how making use of the already existing data as substantial prior information improve the accuracy of prediction, thereby reducing the number of states to be valued which in turn reduces the cost of cross-country valuation. Work on the demonstration of this idea in a smaller country setting is still in progress.

One limitation of this study is that, as many international agencies recommend the use of country own value sets to generate QALYs, it is unclear whether a value set generated using own country data modelled alongside another country’s dataset would be acceptable. However, this may not be a concern if the estimates are accurate and the ordering of health states and location on the 1-0 full health-dead scale is similar to those achieved using a large scale valuation study.

Our basic model Eq. 1 has the potential to allow for more than two countries to be analysed. Additionally, it would be possible to generalize Eqs. 2 and 3 to handle more than two countries. Indeed, we can generalize further to a generic form

$$ E\left(u\left(\mathbf{x}\right)\right)=\sum \limits_{k=1}^nE\left({u}_k\left(\mathbf{x}\right)\right)+\gamma +{\beta}^{\prime}\mathbf{x} $$

and variance-covariance matrix

$$ \sum \limits_{\boldsymbol{k}=1}^{\boldsymbol{n}}\operatorname{cov}\left({u}_k\left(\mathbf{x}\right),{u}_k\left({\mathbf{x}}^{\prime}\right)\right)+{\sigma}^2\mathrm{c}\left(\mathbf{x},{\mathbf{x}}^{\prime}\right) $$

where $\sum \limits_{k=1}^nE\left({u}_k\left(\mathbf{x}\right)\right)$ is the total mean utility of health state x and $\sum \limits_{\boldsymbol{k}=\mathbf{1}}^{\boldsymbol{n}}\operatorname{cov}\left({u}_k\left(\mathbf{x}\right),{u}_k\left({\mathbf{x}}^{\prime}\right)\right)$ is the total variance-covariance matrix between *u*_*k*_(**x**) and *u*_*k*_(**x**′) for two different states **x** and **x**′, all of which are readily available from the analysis of the *n* available countries data.

A final note regarding the potential impact of our study in terms of health and quality of life gains: Note from Table 1 that health state 635,651, for instance, has an estimated health state utility value of 0.3799 from the HK model and 0.4841 from the HK/UK model. Thus, the difference in utility estimates is nearly 0.11. This could bring about an shift in QALYs from a treatment that prolongs life by 1 yr from 0.5 to 0.61. This implies that if a treatment costs 12,000, for example, the cost per QALY would decrease from £24,000 to £19,672, thereby it below the cost effectiveness threshold used by National Institute for Health and Clinical Excellence. In other words, it could influence whether or not a treatment is funded. Heijink et al. [22] found analogous impact of different valuation functions on QALYs.

## Conclusion

In conclusion, this novel method of modelling utility functions permitted the UK data to contribute considerable prior to the HK analysis. Consequently, estimates of the HK utilities for the various SF-6D health states could be generated much more precisely than would have been the case if the data from HK study was used alone. It is likely that this will prove to allow the need for much smaller studies compared to what has been employed when developing valuations for new countries. The promising results suggest that existing preference data could be combined with valuation study in a new country to generate preference weights, making own country value sets more achievable for low and middle income countries.

## Abbreviations

AQoL: Assessment of Quality of Life
EQ-5D: The EuroQol
HK: Hong Kong
HRQoL: Health-related quality of life
HUI: Health Utilities Index
LN(.,.): Log-Normal distribution
MLE: maximum likelihood estimator
MVH: Measurement and Valuation of Health
N(.,.): Normal distribution
QALYs: Quality adjusted life years
QWB: Quality of well-being
RMSE: Root mean square error
SD: Standard deviation
SF-6D: Short form 6 dimensions health survey
SG: Standard gamble
TTO: Time trade-off
UK: United Kingdom
VAS: visual analogue scale
